# Limitations of turbidity process probes and formazine as their calibration standard

**DOI:** 10.1007/s00216-016-9893-1

**Published:** 2016-10-01

**Authors:** Marvin Münzberg, Roland Hass, Ninh Dinh Duc Khanh, Oliver Reich

**Affiliations:** Physical Chemistry – innoFSPEC, University of Potsdam, Am Mühlenberg 3, 14476 Potsdam-Golm, Germany

**Keywords:** Photon Density Wave spectroscopy, Turbidity probes, Formazine, Calibration standard, Process analytical technology

## Abstract

Turbidity measurements are frequently implemented for the monitoring of heterogeneous chemical, physical, or biotechnological processes. However, for quantitative measurements, turbidity probes need calibration, as is requested and regulated by the ISO 7027:1999. Accordingly, a formazine suspension has to be produced. Despite this regulatory demand, no scientific publication on the stability and reproducibility of this polymerization process is available. In addition, no characterization of the optical properties of this calibration material with other optical methods had been achieved so far. Thus, in this contribution, process conditions such as temperature and concentration have been systematically investigated by turbidity probe measurements and Photon Density Wave (PDW) spectroscopy, revealing an influence on the temporal formazine formation onset. In contrast, different reaction temperatures do not lead to different scattering properties for the final formazine suspensions, but give an access to the activation energy for this condensation reaction. Based on PDW spectroscopy data, the synthesis of formazine is reproducible. However, very strong influences of the ambient conditions on the measurements of the turbidity probe have been observed, limiting its applicability. The restrictions of the turbidity probe with respect to scatterer concentration are examined on the basis of formazine and polystyrene suspensions. Compared to PDW spectroscopy data, signal saturation is observed at already low reduced scattering coefficients.

## Introduction

Process analytical technologies (PAT) are important to ensure quality, efficiency, and safety during chemical, physical, and biotechnological processing [1]. In contrast to post-production analysis, these technologies provide information about the operating process and thus allow for direct quality control. More than 80 % of all materials during industrial processing are processed in form of particles [2]. Suspended particles, droplets, or cells in the nano or micrometer size regime often exhibit high turbidities. Furthermore, their processing is often performed at high concentrations of the dispersed phase. A common approach to monitor the process state is implementing a turbidity or optical density probe [3–9]. These probes are, reputedly, easy to use and financially attractive. As stated in the ISO 7027:1999 “Water quality—Determination of turbidity”, for the calibration of such probes, a suspension of formazine has to be used [10]. Based on this international calibration standard, several measurement units are derived, e.g., formazine attenuation units (FAU), formazine nephelometric units (FNU), formazine turbidity units (FTU), and nephelometric turbidity units (NTU). They depend on the field of use and measurement geometry.

The synthesis of formazine is simple [11]. Mixing aqueous solutions of hydrazine sulfate and hexamethylenetetramine in concentrations, as stated in the ISO 7027, creates a suspension with a certain turbidity used to define 4000 FTU [10]. Despite the importance of this calibration standard and its requirement according to the ISO 7027, there is no information available about the optical properties of formazine in the literature. In addition, no characterization of the formation process could be found. Not even a turbidity or optical density probe has been used to monitor the polymerization process of formazine so far. However, despite being easy to prepare, there are some disadvantages of formazine. It is synthesized from a carcinogenic substance (hydrazine sulfate), long-time stability has not been investigated thoroughly, and the overall low turbidity of 4000 FTU limits the calibration range of turbidity probes for process monitoring. As a side note, it has to be stated that the current ISO 7027 contains a fundamental translation error in the German version (cf. “Formazine synthesis” section for details).

Due to the importance of formazine, the robustness of its formation is investigated. The influence of the reaction temperature, concentration of reactants as well as other process parameters (e.g., stirring) needs to be evaluated to understand their influence on the received product in relation to its optical properties and their reproducibility. Besides using a turbidity probe for such investigations, another independent process analytical technology should be applied as reference method. Here, Photon Density Wave (PDW) spectroscopy [12–16] is well suited for this polymerization reaction, since it absolutely and independently quantifies the optical properties of the formazine suspension, i.e., the absorption coefficient (*μ*_a_) and the reduced scattering coefficient (*μ*_s_′). Furthermore, PDW spectroscopy is based on “first principles”; thus, it can be directly related to basic physical quantities including equation-based error estimation. Separating the absorption and the reduced scattering coefficient is important for the optical characterization of any turbid material, which is a challenge for most other (process) analytical techniques [17].

### Formazine

Formazine is a polymer precipitating after mixing aqueous solutions of hydrazine sulfate and hexamethylenetetramine [11]. In a first step, hexamethylenetetramine reacts with sulfuric acid, created by dissolving hydrazine sulfate, to formaldehyde. Afterwards, formazine is created in a condensation polymerization of formaldehyde and the dissolved hydrazine [18]. The reported reproducibility of the received turbidity is about 1 % with a significant temperature influence on the calibration results [11, 18]. However, since continuous stirring during the reaction time is not requested by the ISO 7027, temperature inconsistency might cause problems, if synthesized in bigger volumes.

### Turbidity probes

Different types of turbidity probes are available, which can be differentiated by the measurement principle [18–24]. Typical geometries include transmission (optical density), reflection, and transflection probes. For higher concentrations, reflection probes are more suitable, since they do not depend on a defined optical path length through which light has to travel. In addition, optical cavities as in transmission and transflection probes can clog more easily (“probe fouling”). Therefore, in-line probes are often operating in a reflection or backscatter setting. Light of a certain wavelength or white light is guided into the material and the reflected intensity *I*_R_ is measured at almost 180°. The typical wavelength used for such measurements is (860 ± 30) nm (as requested by the ISO 7027), as the expected light absorption, e.g., in colored systems, is usually low at this wavelength regime. This technique, in general, works well for low turbidities, i.e., low concentrations of particles, cells, or droplets and/or low refractive index differences between the continuous and the dispersed phase.

### Photon Density Wave spectroscopy

PDW spectroscopy is a fiber-based in-line measurement technology to independently determine the absorption and reduced scattering coefficient of a liquid suspension. Intensity-modulated laser light is coupled into the multiple light scattering material via an optical fiber, creating a photon density wave. The change in amplitude and phase of the PDW due to the interaction of light with the material is characterized with a second fiber as function of modulation frequency and detector to emitter fiber distance. The independent determination of absorption and scattering is based on multiple scattering and absorption of photons inside the material [25, 26]. This limits the applicability of this technique for investigating low concentrations since a multiply light scattering dispersion is required, i.e., the material has to be rather turbid.

## Experimental

### Formazine synthesis

Hydrazine sulfate and hexamethylenetetramine (both Sigma-Aldrich Chemie GmbH, Taufkirchen, Germany) were separately dissolved in purified water (Milli-Q, Merck KGaA, Darmstadt, Germany) to produce stock solutions of 10 and 100 g L^−1^, respectively. For the synthesis of formazine, 300 mL of each stock solution was mixed in a jacketed beaker (1200 mL, Neubert Glas GbR, Geschwenda, Germany) to generate a formazine suspension of 4000 FTU. The temperature of the reaction mixture was controlled by a thermostat (Ministat 230w, Huber Kältemaschinenbau GmbH, Offenburg, Germany) equipped with an external temperature sensor.

According to the ISO 7027, the English version correctly states “Quantitatively pour the two solutions into a 100.0 mL volumetric flask, dilute to the mark with water and mix well.” In contrast, the German version asks for “Die beiden Lösungen in je einen 100,0 mL Kolben geben, mit Wasser bis zur Marke auffüllen und gut mischen.,” which might be translated to “Pour both solutions into a 100.0 mL flask *each*, fill with water to the mark and mix well.” In fact, all turbidity probes calibrated according to the German ISO should therefore measure off by a factor of two. The “solutions” are to be prepared identically in each language version.

Despite the ISO 7027 regulation, the reacting solution was stirred, if not stated otherwise, continuously during formation by a magnetic stirrer (Hei-End, Heidolph Instruments GmbH & Co. KG, Schwabach, Germany) at 200 rpm with a triangular stirring bar. The relative concentrations *c*_rel_ of the stock solutions were varied systematically to 5, 50, 75, 125, 150, 200, 275, and 333 % of the stock solution described above (i.e., 100 %), corresponding hyptothetically to 200, 2000, 3000, 5000, 6000, 8000, 11000, and 13320 FTU, respectively. Here, 333 % is the maximal concentration due to the solubility of hydrazine sulfate in water at 25 °C. The total reaction volume was kept constant at 600 mL during all experiments. Both process analytical technologies were implemented simultaneously in the reaction vessel.

### Polystyrene suspension

An aqueous polystyrene suspension (Fraunhofer Institute for Applied Polymer Research, Potsdam, Germany) as material with higher turbidity than formazine was also characterized. The particle dimensions are: dynamic light scattering: hydrodynamic diameter *d*_h_ = 364 nm, polydispersity index PDI = 0.037 (z-average, Zetasizer Nano ZS, Malvern Instruments Ltd, Worcestershire, UK); static light scattering: geometrical diameter *d*_g_ = (362 ± 52) nm (LS 13 320, Beckman Coulter GmbH, Krefeld, Germany); electron microscopy: geometrical diameter *d*_g_ = (342 ± 8) nm (175 particles manually analyzed, Quanta 250 with STEM-detector, FEI, Eindhoven, Netherlands). The solid content was 7.34 % measured by dry weighing. The volume fraction was calculated based on the density of water (*ρ* = (0.9972 ± 0.0001) gcm^−3^ at 20 °C, DM40, Mettler Toledo GmbH, Gießen, Germany) and polystyrene (*ρ* = 1.055 g cm^−3^ [27]). Different volume fractions were produced by dilution with purified water.

### Turbidity probe

For the turbidity measurements, a fiber-optical turbidity probe (inPro8200, Mettler Toledo GmbH, Gießen, Germany) in combination with a spectrometer (MCS600 with light source CLD 600 and detectors MCS 621 and MCS 611, Carl Zeiss AG, Jena, Germany) was used. The probe was installed vertically. The probe tip was positioned at half beaker radius and at half filling level, i.e., at 300 mL. For data analysis, the wavelength of 860 nm was chosen because of its utilization in other commercial setups and its request according to the ISO 7072. The integration time was about 100 ms, and every 5 s a data point was measured.

### Photon Density Wave spectrometer

The Photon Density Wave spectrometer is self-built, including lasers at eight different wavelengths (515, 637, 690, 751, 778, 906, 940, and 982 nm). It consists of a vector network analyzer (ZVA8, Rohde & Schwarz GmbH & Co. KG, Munich, Germany) for intensity modulation at different frequencies and signal analysis and an avalanche photo diode (APM-400P, Becker & Hickl GmbH, Berlin, Germany) for detection. The emission and detection fiber tips were positioned around the center of the beaker at half filling level. More technical and theoretical details on Photon Density Wave spectroscopy are described elsewhere [26, 28]. For data analysis, the index of refraction of the suspension at the experimental wavelength is needed. For the polystyrene suspension, it can be calculated [12] based on the refractive indices of pure water and polystyrene [29]. For the formazine suspension, refractive indices of the pure polymer are still missing. Here, 1.45 and 1.44 were anticipated for the wavelengths of 690 and 906 nm, respectively.

## Results and discussion

### Standard synthesis of formazine and influence of stirring

**Fig. 1:**
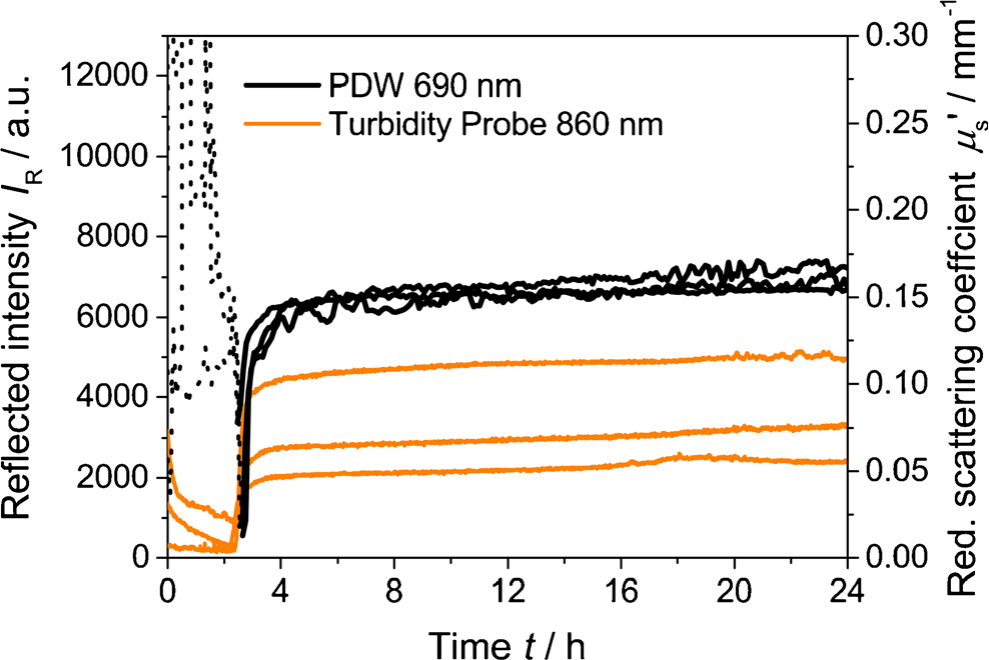
Reflected intensity at 860 nm and reduced scattering coefficient at 690 nm as function of time for three repeated syntheses of formazine at 25 °C, with stirring at 200 rpm. *Dotted line* indicates signal noise from PDW spectroscopy due to insufficient light scattering

Figure 1 displays three repeated syntheses of formazine at 100 % relative concentration and 25 °C (i.e., 4000 FTU). Here, the formation of formazine occurs approximately 2.5 h after initial mixing of the stock solutions, leading to a turbid suspension. The sharp increase of turbidity at this point in time is assumed to indicate the beginning of the precipitation of formazine.

After approximately 5 h, the reduced scattering coefficient as well as the reflected intensity *I*_R_ reached a plateau. The reduced scattering coefficient indicates a reproducibility of about 4 % (single standard deviation) at 24 h, whereas the reflected intensity at this time, obtained by the turbidity probe, varies by factor of approximately 2. This strongly disagrees with the stated reproducibility of formazine suspensions of about 1 % [11, 18].

Since PDW measurements indicate that formazine can be reproducibly formed, it is likely that the turbidity probe measurements are highly dependent on the surrounding conditions. Even though the probe was implemented in the reaction vessel as reproducible as possible, surface reflections from the jacketed beaker, stirrer bar, etc. seem to affect the obtained reflected intensities. Additionally, also the surrounding light has an influence on the detected reflected intensities for the chosen experimental setup. Both effects not only cause problems while calibrating such turbidity probes, but also limit the applicability of these probes in any process environment, since the effect of the measurement geometry and other external influences is significant. Due to the inconsistency of the reflected intensities for the replicated formazine formations, all turbidity data are given in arbitrary units and are not expressed (“calibrated”) in FTUs.

Comparing the precipitation onset, a different starting time between the turbidity probe and PDW spectroscopy can be noted. The delay of the reduced scattering coefficient in comparison to the turbidity probe is about 15 to 25 min in Fig. 1. This is caused by a certain minimal turbidity level needed for successful PDW experiments, i.e., material exhibiting multiple light scattering and therefore requiring a higher scatterer concentration. Accordingly, before the polymer precipitation sets in at approximately 2.5 h, only signal noise is obtained by PDW spectroscopy (Fig. 1, dotted lines). These false signals are identified by extremely large deviations between experimentally obtained amplitude and phase of the PDW and their fits during data analysis. Hence, data points are discarded for *χ* ^2^ values of >100 (sum of the squared deviations between experimental data and fit) [26]. For the turbidity probe, also trends are observed before the precipitation onset, probably again due to the above-stated geometric effects and external light sources.

**Fig. 2:**
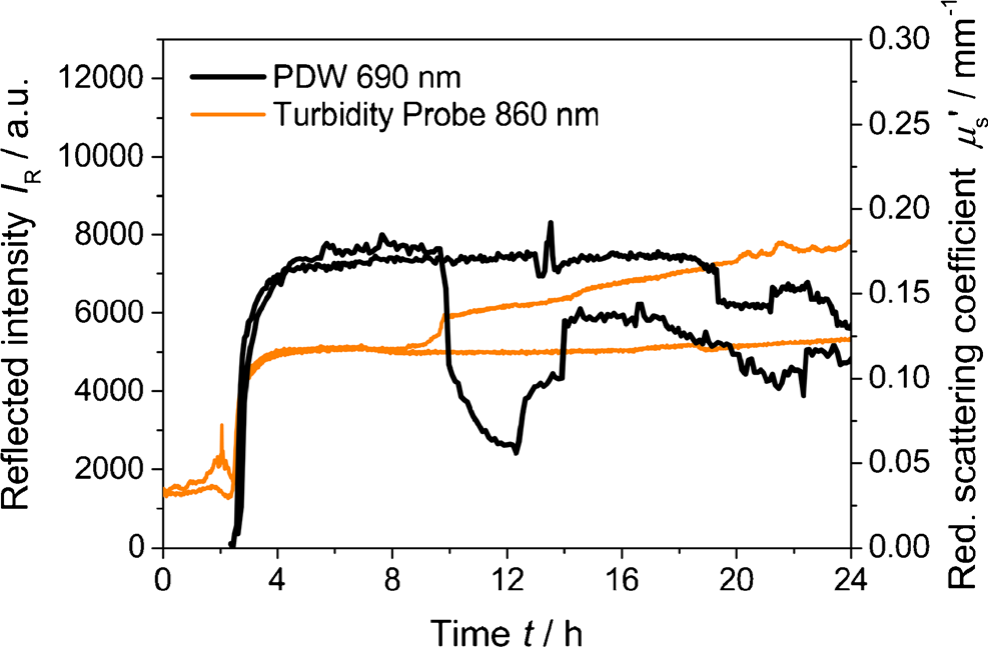
Reflected intensity at 860 nm and reduced scattering coefficient at 690 nm as function of time for two syntheses of formazine at 25 °C, without stirring

In the ISO 7027, it is described that the formazine suspension should be prepared without stirring over 24 h after initial mixing. Figure 2 displays two repeated syntheses without stirring. While the precipitation onset time and initial values of *μ*_s_′ and *I*_R_ are similar to the data shown in Fig. 1, strong variations are detected after around 10 h, which are very likely related to sedimentation effects.

**Fig. 3:**
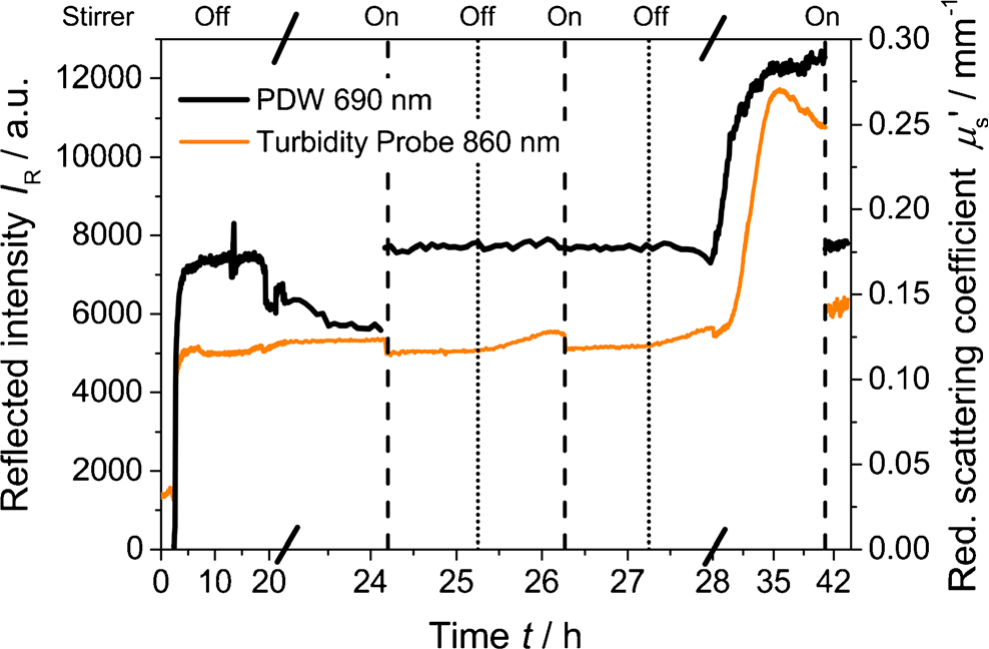
Reflected intensity at 860 nm and reduced scattering coefficient at 690 nm for a synthesis of formazine at 25 °C without stirring for the first 24 h. Afterwards, the stirrer was alternately turned on (*dashed line*) and off (*dotted line*)

The influence of stirring is shown in Fig. 3, where the stirrer was switched on and off alternatingly in one of the batches from Fig. 2 after 24 h. Immediately after stirring for the first time, the partly sedimented suspension is resuspended and constant reduced scattering coefficients and reflected intensities at the expected levels are measured. In contrast, stopping stirring reinitiates sedimentation, with pronounced effects on both optical probes for longer settling times (e.g., after 28 h in Fig. 3). But also after long settling times resuspension seems feasible (stirrer on at 42 h in Fig. 3). However, the absolute values of *μ*_s_′ and *I*_R_ here (0.179 mm^−1^ at 690 nm and 6.3 10^3^ at 860 nm, respectively) differ from the ones at the initial plateau period (0.169 mm^−1^ at 690 nm and 5.1 10^3^ at 860 nm).

If resuspension is not fully reproducible or if the polymer undergoes permanent alteration remains unclear. However, despite the regulations stated in the ISO 7027, the experimental observations indicate that the synthesis of formazine should be performed under continuous stirring, allowing also for better temperature control. Furthermore, it has to be stressed that any protocol for a turbidity probe calibration with formazine should include its controlled resuspension. All further syntheses of formazine presented in this work were realized under stirring.

### Temperature dependence of formazine synthesis

**Fig. 4:**
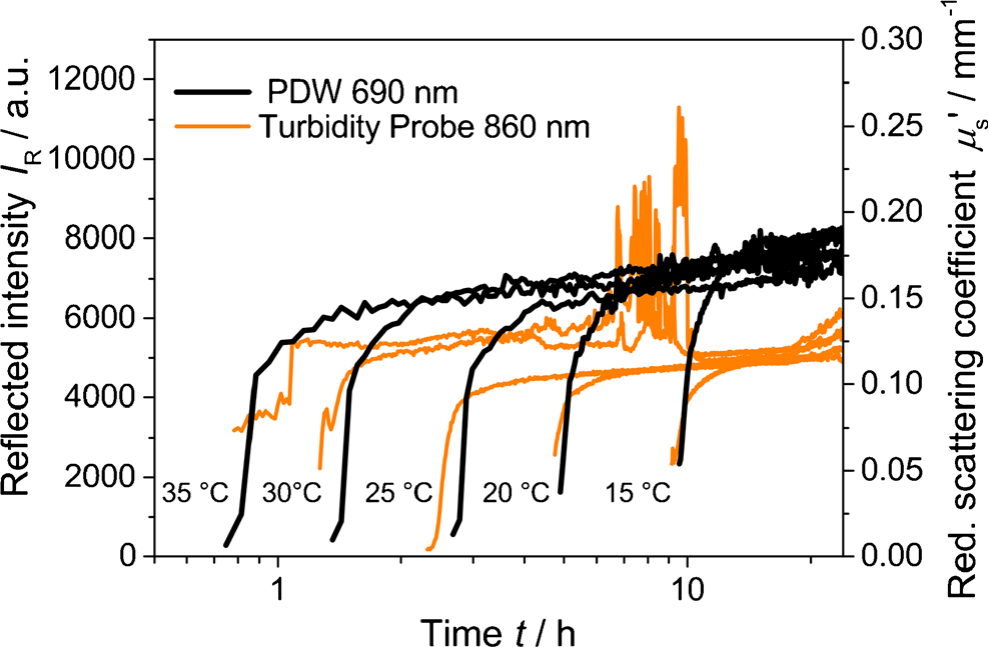
Reflected intensity at 860 nm and reduced scattering coefficient at 690 nm for formazine syntheses at different temperatures, with stirring at 200 rpm

The ISO 7027 requests a reaction temperature of (25 ± 3) °C. Besides homogenous temperature distribution within the reaction vessel (stirring), the supposedly long reaction time of 24 h implies active temperature control. To evaluate the temperature influence on the formazine synthesis, the reaction temperature was varied systematically (Fig. 4).

Increasing temperatures cause significantly earlier onsets of the formazine precipitation, observed by both measurement technologies. At 24 h, the obtained experimental values of *I*_R_ and *μ*_s_′ vary. The standard deviation in Fig. 4 at 24 h is approximately 4 % for *μ*_s_′ without clear temperature dependence (data not shown). In contrast to turbidity measurements, PDW spectroscopy is not affected by ambient light. Therefore, the variation of 4 % in *μ*_s_′, also seen in repeated measurements at the same temperature, is attributed mostly to the reproducibility of the formazine synthesis itself. However, limitations of reproducibility within the experimental setup may still have an influence on PDW spectroscopy. For the turbidity probe trends, very high noise is obtained at around 8 h. This is caused by sunlight entering the lab during sunset. Similar effects are observed 12 h later (sunrise), where again *I*_R_ is affected. For the chosen turbidity measurement setup, this clearly indicates how substantially such experiments can be biased by external influences.

**Fig. 5:**
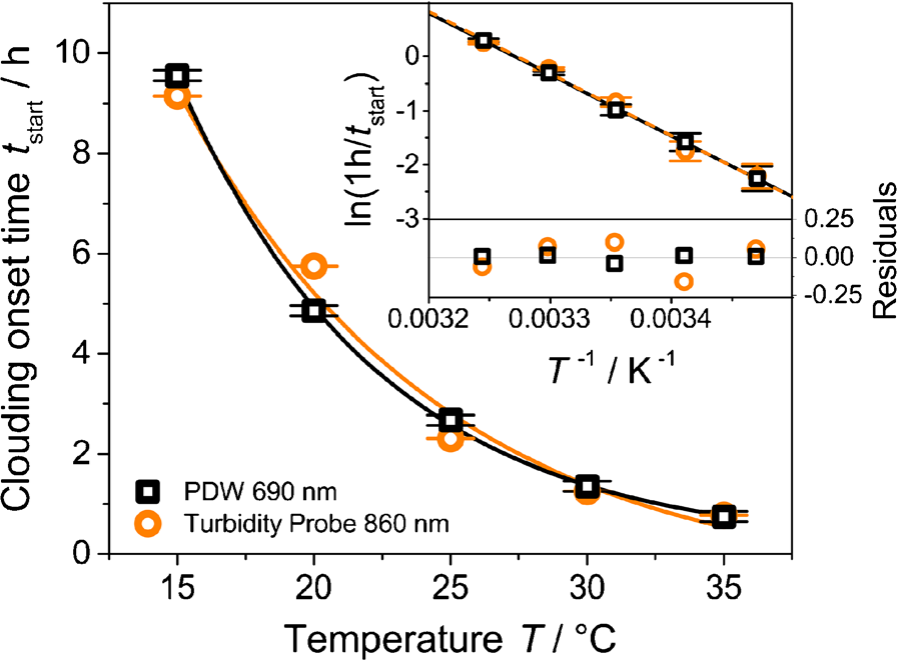
Clouding onset time from Fig. 4 as function of reaction temperature with exponential fits and linearization with an Arrhenius approach (*inset*) as well as its residuals

Figure 5 displays the clouding onset time of the formazine precipitation (*t*_start_) as function of the reaction temperature. An exponential decay is observed, indicating that the clouding onset represents a certain reaction state during the formazine synthesis. Linearization based on an Arrhenius approach yields a remarkable linearity (Fig. 5 inset). The activation energy calculated from the slopes is (94.0 ± 1.2) kJ mol^−1^ (by PDW spectroscopy) and (95.2 ± 5.8) kJ mol^−1^ (by turbidity measurement). These values are close to the typical range for condensation reactions of formaldehyde (50–83 kJ mol^−1^ [30, 31]).

### Concentration dependence of formazine synthesis

**Fig. 6:**
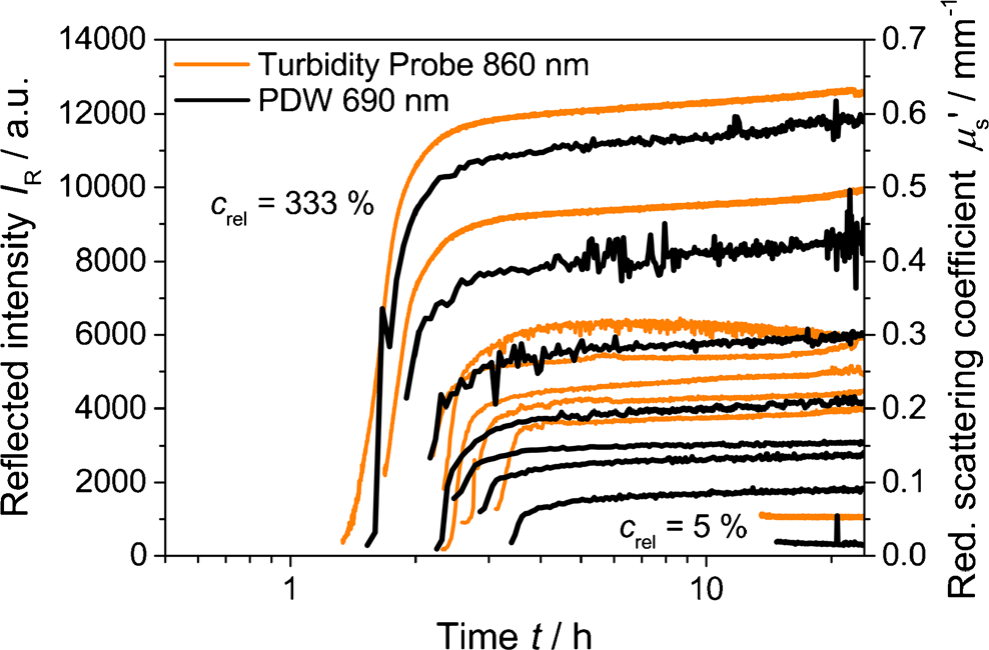
Reflected intensity at 860 nm and reduced scattering coefficient at 690 nm for formazine syntheses with different relative starting concentrations

Figure 6 displays *μ*_s_′ and *I*_R_ as function of time for a simultaneous variation of the relative concentration of the reactants, ranging from 5 to 333 %. Increasing concentrations result in larger experimental values for *μ*_s_′ and *I*_R_ at 24 h.

**Fig. 7:**
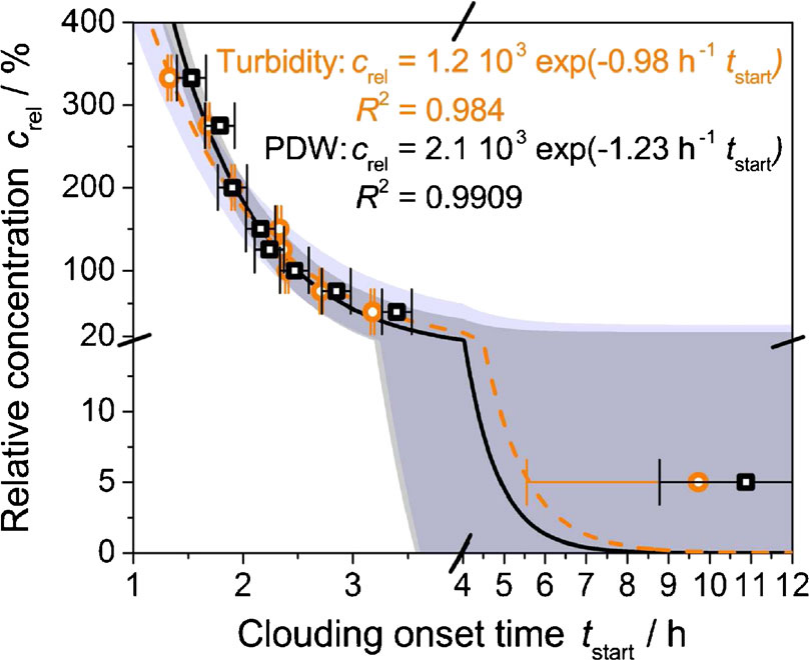
Relative stock solution concentration as function of the clouding onset time from Fig. 6 with fits and resulting 95 % confidence intervals

In addition, it also can be noted that the clouding onset time varies systematically, with earlier formazine precipitation at higher concentrations (Fig. 7). The experimental relation of *c*_rel_ vs. *t*_start_ can be described by an exponential decay function of the form *c*_rel_ = A exp(−B *t*_start_). Including an additional *y*-axis intercept could yield the solubility of formazine in water at 25 °C. However, its precise determination would require experiments at low concentrations (e.g., *c*_rel_ = 5 % and lower). For the fits given in Fig. 7, the data set of *c*_rel_ = 5 % has not been used due to the significant error in the associated value of *t*_start_ at that concentration for both experimental methods. Besides the experimentally obtained concentration dependence of the clouding onset time, it remains to be discussed what further type of kinetic information can be obtained from the concentration influence.

**Fig. 8:**
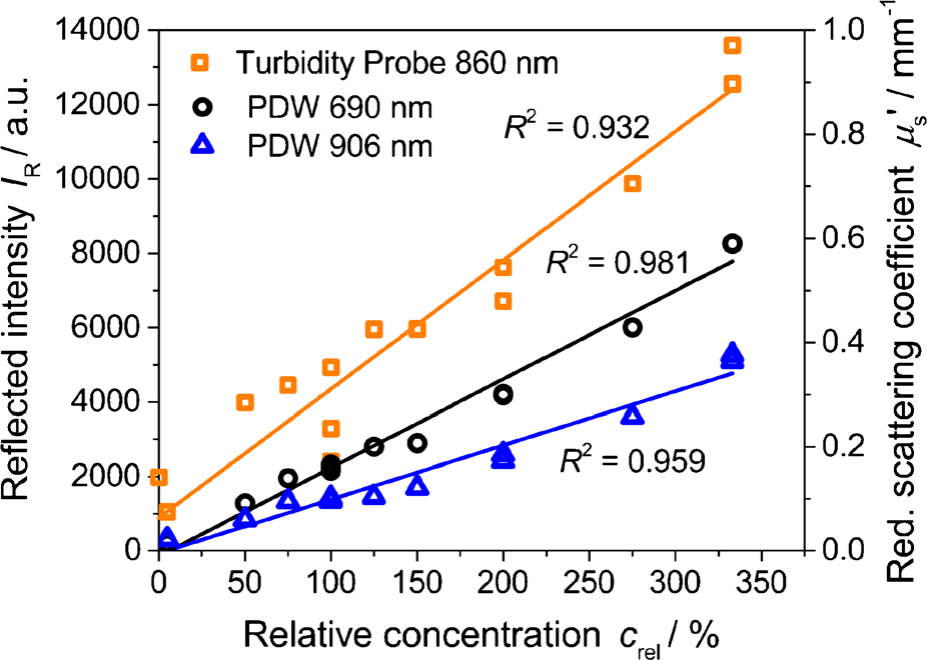
Reflected intensity at 860 nm (*squares*) and reduced scattering coefficient at 690 nm (*circles*) and 906 nm (*triangles*) after 24 h for different relative concentrations with linear fits, including repeated experiments at c_rel_ = 100, 200, and 333 %. Relative intensity for pure water is not included into the fit

In contrast, the experimental data obtained at 24 h show a linear relation with changing relative concentration (Fig. 8). Since the slope of *μ*_s_′ as function of concentration is strongly affected by particle size, changes in particle dimension would lead to a non-linear behavior, even for moderate particle concentrations investigated here [12, 26]. Thus, the linearity suggests that only the concentration of formazine particles, but not their structure or size is affected. In Fig. 8, PDW spectroscopy data is also shown for wavelengths of 690 and 906 nm for a first spectral evaluation. Even though the overall fit quality is low (e.g., compare to Fig. 10, with polystyrene as scatterer), some conclusions can be drawn. For PDW spectroscopy, repeated experiments result in similar reduced scattering coefficients and therefore good reproducibility of the formazine synthesis. Furthermore, for PDW spectroscopy, the obtained *y*-axis intercept is close to zero ((−0.0106 ± 0.0133) mm^−1^ for 690 nm and (−0.0051 ± 0.0120) mm^−1^ for 906 nm).

This is not the case for the turbidity data. Here, a significant *y*-axis intercept is noted (*I*_R_(*c*_rel_ = 0) = 9.8 10^2^), with *I*_R_ = 2.0 10^3^ for clear water being even higher (this data point was not included in the linear regression). In addition, the poor reproducibility of *I*_R_ trends (cf. Fig. 1) is also noted at other concentrations. On the contrary, the chosen turbidity probe can proportionally measure the influence of concentration up to the maximal possible concentration of *c*_rel_ = 333 %. With respect to the ISO 7027, this indicates that also higher FTUs than 4000 (i.e., *c*_rel_ = 100 %) could be realized for instrument calibrations on the basis of formazine.

The experimentally obtained mean absorption coefficient from PDW spectroscopy for all relative concentrations was determined to approximately (5.4 ± 2.9) 10^−4^ mm^−1^ and (6.2 ± 2.9) 10^−3^ mm^−1^ for 690 and 906 nm, respectively, exhibiting no systematic tendencies (data not shown). They are in the range of the absorption coefficients of pure water at these wavelengths (cf. Fig. 11).

### Quantification of high turbidities

Heterogeneous chemical, physical, or biotechnological processes quite often exhibit much stronger light scattering than can be represented by formazine as calibration standard. For example, at *c*_rel_ = 333 % PDW spectroscopy measures reduced scattering coefficients of less than 1 mm^−1^ at various wavelengths (cf. Fig. 8). In reality, experimental values of more than 1000 mm^−1^ have been obtained (e.g., for aqueous TiO_2_ suspensions, data not shown). To evaluate to what extent turbidity process probes can address also higher turbidities, i.e., at which values a signal saturation for *I*_R_ sets in, further concentration series with polystyrene suspensions have been performed.

**Fig. 9:**
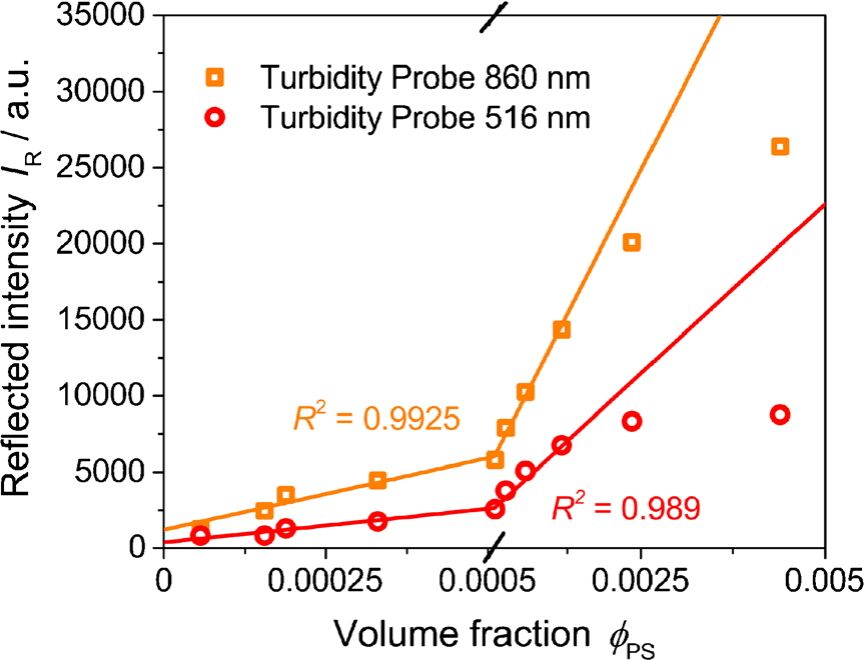
Reflected intensity at 516 nm (*circles*) and 860 nm (*squares*) as function of volume fractions for a polystyrene suspension

Figure 9 shows reflected intensities at two different wavelengths for a polystyrene suspension at volume fractions below 0.005. Already above very low concentrations (volume fraction *Φ*_PS_ of approximately 0.0014), a deviation from the linear fit can be noted for both wavelengths. At this concentration, the turbidity signal starts to saturate (for the chosen polystyrene suspension). With respect to the reflected intensity, this saturation starts at around 1.7 10^4^ for a wavelength of 860 nm (max. *I*_R_ for formazine at *c*_rel_ = 333 % was 1.2 10^4^ at 860 nm, cf. Fig. 8). In comparison to Fig. 8, a linear detection range can clearly be determined, limiting the use of turbidity probes in highly turbid systems.

**Fig. 10:**
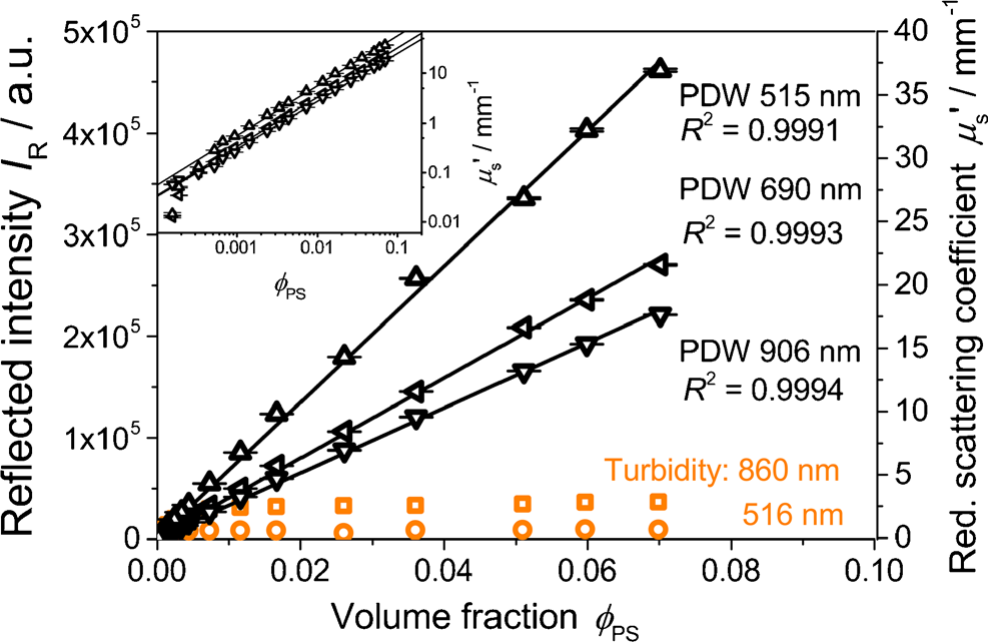
Reflected intensity at 516 nm (*circles*) and 860 nm (*squares*) and reduced scattering coefficient at 515, 690, and 906 nm (*triangles*) as function of volume fraction for a polystyrene suspension. *Inset* displays the reduced scattering coefficients in a double-logarithmic plot

Accordingly, the turbidity probe is insensitive to higher concentrations (Fig. 10). Here, results from the turbidity probe are compared with PDW spectroscopy in a way that the individual linear regressions are mostly in parallel for very low concentrations (by *y*-axis scaling). As can be seen from PDW spectroscopy measurements, *I*_R_ values of up to 4.5 10^5^ at 516 nm should be expected for the highest polystyrene volume fraction. Instead, *I*_R_ saturates at approximately 1 10^4^ at that wavelength. In contrast, PDW spectroscopy provides increasing reduced scattering coefficients over the entire investigated concentration range. It has to be stressed that the increase in *μ*_s_′ is actually non-linear towards higher volume fractions, which can be described by so-called dependent light scattering and which is a material property, not a measurement limitation [26]. While high turbidities do not limit the application space of PDW spectroscopy, the theoretical requirement of *μ*_s_′ > > *μ*_a_ [26] at the experimental wavelength is critical for low turbidities. The inset in Fig. 10 displays the experimental *μ*_s_′ for the polystyrene concentration series in double-logarithmic scale. As anticipated, deviations occur towards very low particle concentrations. For the investigated suspension, a lower measurement range limit for the reduced scattering coefficient of approximately 0.05 mm^−1^ can be identified which translates here to a polystyrene volume fraction of approximately 1.6 10^−4^. However, for quite a number of processes investigated with PDW spectroscopy, too low turbidity has not been of relevance [12, 13].

For practical considerations, the obtained findings imply that the saturation level of a turbidity sensor should always be estimated in order to define the suitable concentration range. This has to be performed with the material under investigation or at least with materials of equivalent optical properties. In contrast, for PDW spectroscopy as process analytical technology, a certain degree of turbidity is always needed. Though a threshold of approximately 0.05 mm^−1^ for *μ*_s_′ was found, this value may be different if significant light absorption occurs at the experimental wavelength used.

**Fig. 11:**
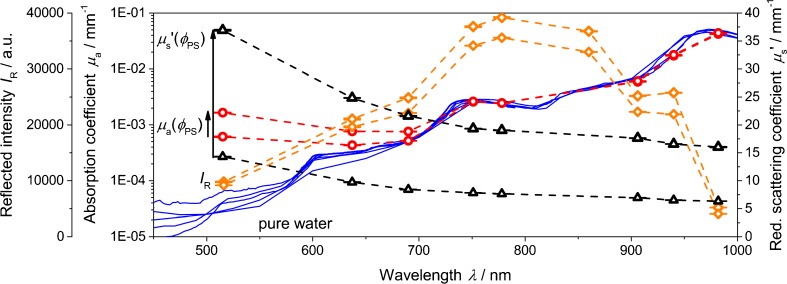
Reflected intensity (*diamonds*), absorption coefficient (*circles*), and reduced scattering coefficient (*triangles*) as function of wavelength for volume fractions of 0.07 and 0.025 of a polystyrene suspension, plus absorption coefficient for pure water [32–39]

The calibration-free and wavelength-dependent separation of light absorption and light scattering is a fundamental benefit of PDW spectroscopy. Figure 11 displays reflected intensities from the turbidity probe and absorption as well as reduced scattering coefficients from PDW spectroscopy as a function of wavelength for two polystyrene concentrations. In addition, absorption coefficients of pure water [32–39] are shown.

As anticipated, the reduced scattering coefficient increases with higher concentration and with lower wavelength. Such a trend is not found in the reflected intensities. Besides the saturation effect, the spectrum represents the emission/detection characteristics of the light source and the detector within the spectrometer. To measure the typical increase of light scattering towards smaller wavelengths, the experimental turbidity setup would need to be calibrated with respect to the light source, detector, and all other optical elements (e.g., fibers). For practical consideration, this may cause additional concerns (e.g., aging of the light source, constant light coupling into the fiber-optical cables, wavelength-dependent light losses due to fiber bending, etc.). In contrast, the wavelength-dependent reduced scattering coefficient is very helpful for process monitoring in systems of particles, droplets, or cells where concentration and size varies simultaneously. As a consequence, besides the saturation and calibration problem of a turbidity probe, multiwavelength measurements are highly recommended for turbidity measurements as well as for PDW spectroscopy.

The absorption coefficient in Fig. 11 scales over three orders of magnitude for the aqueous polystyrene suspension. Particularly for the wavelength range above 700 nm, the experimental values approach the absorption coefficient of pure water. However, below 700 nm, a higher absorption than pure water is observed, which is attributed to the increasing concentration of organic material in the suspension.

**Fig. 12:**
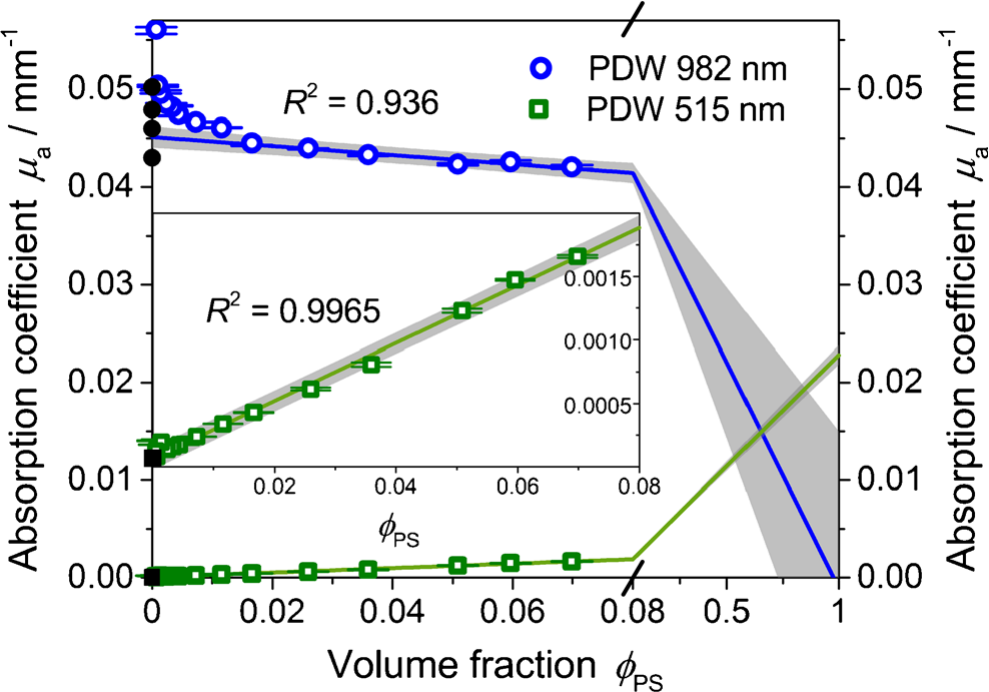
Absorption coefficient at 515 nm (*squares*) and 982 nm (*circles*) as a function of volume fractions of a polystyrene suspension and absorption coefficient of pure water at *Φ*_PS_ = 0 for both wavelengths (*full symbols*) [32–39]. Deviating absorption coefficients at low volume fractions are neglected for the fit. 95 % confidence interval resulting from fit. *Inset* shows the fitted values at 515 nm in detail

The concentration dependence of the absorption coefficient at 515 and at 982 nm is shown in Fig. 12 in more detail. For not too small absorption coefficients, a linear relation with the volume fraction *Φ*_PS_ is found as anticipated. Extrapolating the linear trends to zero volume fraction (i.e., pure water), absorption coefficients of (0.0451 ± 0.0003) mm^−1^ at 982 nm and (6.3 10^−5^ ± 1.5 10^−5^) mm^−1^ at 515 nm are found. They are in good agreement to the literature data [32–39], as can be also seen in Fig. 12. The non-linear deviation towards larger absorption coefficients at very low volume fractions is regarded as a measurement artifact. At these low concentrations, the required condition *μ*_s_′ > > *μ*_a_ is not fulfilled. Interestingly, this deviation from linearity occurs already at higher volume fractions for the wavelength of 982 nm in comparison to 515 nm. This can be explained by the significantly higher light scattering at 515 nm (cf. Fig. 11), allowing for the determination of absorption coefficients also in very low concentrated systems.

Extrapolating the linear trends in Fig. 12 to a volume fraction of 1 (i.e., theoretically pure polystyrene), absorption coefficients of (−0.0011 ± 0.0063) mm^−1^ at 982 nm and (0.0228 ± 0.0005) mm^−1^ at 515 nm are found. However, since absorption reference data for the vis/NIR region for polystyrene seems not to be available, here it is only referred to the organic fraction within the aqueous polystyrene suspension.

### Considerations about calibration standards

Based on the concentration dependence as observed by PDW spectroscopy for the formazine suspension (cf. Fig. 8), changing the relative target concentration by 1 % causes a shift of the reduced scattering coefficient of 1.07 % at 690 nm and of 1.05 % at 906 nm. Similarly, having the concentration 5 % off the target induces a change of 5.33 % at 690 nm and of 5.26 % at 906 nm in *μ*_s_′. With respect to repeatability, for the three trials investigated here, as well as for syntheses at different temperatures ±4 % from the average *μ*_s_′ (single standard deviation) was obtained at 24 h. For the turbidity probe implemented here, the influence of the surrounding conditions caused severe problems in the repeatability, accounting to deviations of more than ±36 % at 24 h. Therefore, the reproducibility of the formazine synthesis itself is not the limiting factor for quantitative measurements.

For the polystyrene suspension investigated here, much stronger light scattering is observed than what can be achieved with a formazine suspension (e.g., factor of approximately 35 for *μ*_s_′ at 690 nm). In addition, polystyrene provides the advantage of changing the slope of *μ*_s_′(*Φ*_PS_) by adjusting its particle size. This is of benefit if calibration standards with different turbidity dynamics are required. Since even at maximal relative concentration of *c*_rel_ = 333 % the formazine suspension exhibits reduced scattering coefficients of only approximately 0.6 mm^−1^ (depending on the wavelength), it is of very limited use for calibrating probes for the application in concentrated heterogeneous processes. Here, far more turbid calibration material is needed. Though the reproducible production of such material may be more complex (e.g., providing polystyrene particles with always the same particle size distribution), its optical certification may be helpful. In particular, separating light absorption and light scattering, as it is achieved, e.g., by the calibration-free approach of PDW spectroscopy, would allow for new calibration materials. Instead of requiring materials with highly reproducible formation protocols, nearly any dilutable turbid suspension could act as reference material, as long as its optical properties are characterized.

## Conclusion

For the first time, the formazine formation process has been characterized by process analytical technologies. By systematically testing on influences of temperature and concentration, it is found that temperature does not affect the reduced scattering coefficient towards the end of the synthesis. However, the start of formazine precipitation is significantly fastened at higher temperatures, indicating an Arrhenius activation energy of (94.0 ± 1.2) kJ mol^−1^. Changing the reactant concentrations in parallel, a linear relation for the generated turbidity is found. This indicates that mainly the concentration, but not the dimensions, of the formazine structures is affected. Furthermore, based on formazine suspensions, a much larger turbidity range (at least a factor of two) would be available for the calibration of turbidity probes. For the concentration as requested by the ISO 7027, the optical coefficients of the formazine suspension were reproducibly determined by PDW spectroscopy at 690 and 906 nm (*μ*_s_ *′* (0.161 ± 0.006) mm^−1^ and (0.099 ± 0.003) mm^−1^ and *μ*_a_ (5.4 ± 2.9) 10^−4^ mm^−1^ and (6.2 ± 2.9) 10^−3^ mm^−1^, respectively).

In contrast, the turbidity probe measurements indicated very significant problems for reproducible data generation due to surrounding conditions like external light sources or reactor vessel reflections. Although this probe-based technology could linearly measure up to the maximal possible concentration (*c*_rel_ = 333 %) of the formazine suspension, this material still provides only a limited turbidity of *μ*_s_′ < 1 mm^−1^ (in the NIR regime). For more turbid systems, as they do often occur in scientific and industrial reality, the turbidity probe signal trends already into saturation at volume fractions of approximately 0.0014 (for the polystyrene suspension characterized here). For such systems, process analytical technologies being suitable for highest turbidities like Photon Density Wave spectroscopy are the method of choice. Its benefit, the calibration-free and independent determination of the absorption and the scattering properties, as actually favored by the ISO 7027, allow for an improved insight into heterogeneous chemical, physical, or biotechnological processes.
